# Supplementary material for: Megadalton-sized Dityrosine Aggregates of α-Synuclein Retain High Degrees of Structural Disorder and Internal Dynamics
Source: J Mol Biol. 2020 Dec 4;432(24):166689. doi: 10.1016/j.jmb.2020.10.023 (PMC7779668; doi:10.1016/j.jmb.2020.10.023)

Supplementary Information for

Megadalton-sized dityrosine aggregates of α-synuclein retain high degrees of structural disorder and internal dynamics.

Silvia Verzini, Maliha Shah, Francois-Xavier Theillet, Adam Belsom, Jan Bieschke, Erich E. Wanker, Juri Rappsilber, Andres Binolfi and Philipp Selenko

- **Supplementary Figure Legends S1-S3**
- **Supplementary Figures S1-S3**

Supplementary Figure Legends:

Figure S1: Mass spectrometry and NMR analysis of PICUP HMWAs. (a) Representative fragmentation spectra of αSyn dityrosine peptide-pairs following photo-induced crosslinking and proteolytic digestion. Matched αSyn fragments were identified via automated database searches using the Xi software package. **(b)** Overlay of 2D ^{1}H-^{15}N NMR spectra of ^{15}N isotope-labeled, N-terminally acetylated, monomeric αSyn (50 μM, black) and of PICUP-HMWAs (50 μM, red). Resonance signals of N-terminal αSyn residues 1-10 are shown in blue. HMWA resonances broadened beyond detection are labeled, NMR signals of Y39, Y125, Y133 and Y136 are highlighted in grey. NMR spectra were recorded at 283 K (10 °C).

Figure S2: HMWAs in crowded environments. (a) Overlay of 2D ^{1}H-^{15}N NMR spectra of ^{15}N isotope-labeled, N-terminally acetylated, monomeric αSyn (50 μM) in the presence of 200 mg/mL BSA (black). N-terminal αSyn residues 1-10 in the reference spectrum are broadened beyond detection due to transient interactions with BSA. Y39 signal intensity of monomeric αSyn is attenuated for the same reason. The corresponding *in situ* NMR spectrum of HMWAs (50 μM) formed in the presence of BSA is overlaid in red. Broadened HMWA resonances are labeled, NMR signals of Y39, Y125, Y133 and Y136 are highlighted in grey. **(b)** Western blot analysis of RCSN3 lysate recovery times following DEPC treatment to determine compound decomposition. After incubation with DEPC, αSyn (50 μM) and H_2O_2 (10 mM) were added to lysates after 2 h, 4 h, 6 h and 22 h. Residual DEPC activity was monitored via peroxidative crosslinking of αSyn. **(c)** Overlay of 2D ^{1}H-^{15}N NMR spectra of ^{15}N isotope-labeled, N-terminally acetylated, monomeric αSyn (50 μM) in DEPC-treated RCSN3 lysate (3 mg/mL total protein concentration, black) and *in situ* NMR spectrum of HMWAs (50 μM) formed after addition of cyt *c* (50 μM) and 50 mM H_2O_2 (red). HMWA

resonances broadened beyond detection are labeled. Chemical shift changes of N-terminal αSyn residues 1-10 are shown in blue. NMR signals Y39, Y125, Y133 and Y136 are highlighted in grey. All NMR spectra were recorded at 283 K (10 °C).

Figure S3: αSyn and HMWA SUV-binding. (a) Overlay of 2D ^{1}H-^{15}N NMR spectra of ^{15}N isotope-labeled, N-terminally acetylated, monomeric αSyn (50 μM, black) and in the presence of small unilamellar vesicles (SUV) prepared from pig-brain polar lipid extract. Non-broadened NMR signals of detectable C-terminal residues 106-140 are labeled. Y125, Y133 and Y136 are highlighted in grey. **(b)** Overlay of 2D ^{1}H-^{15}N NMR spectra of ^{15}N isotope-labeled HMWAs (50 μM, black) and upon binding to SUVs (red). Non-broadened NMR signals are labeled. Spectra were recorded at 303 K (30 °C).

Figure S1

Verzini *et al.*

(a)

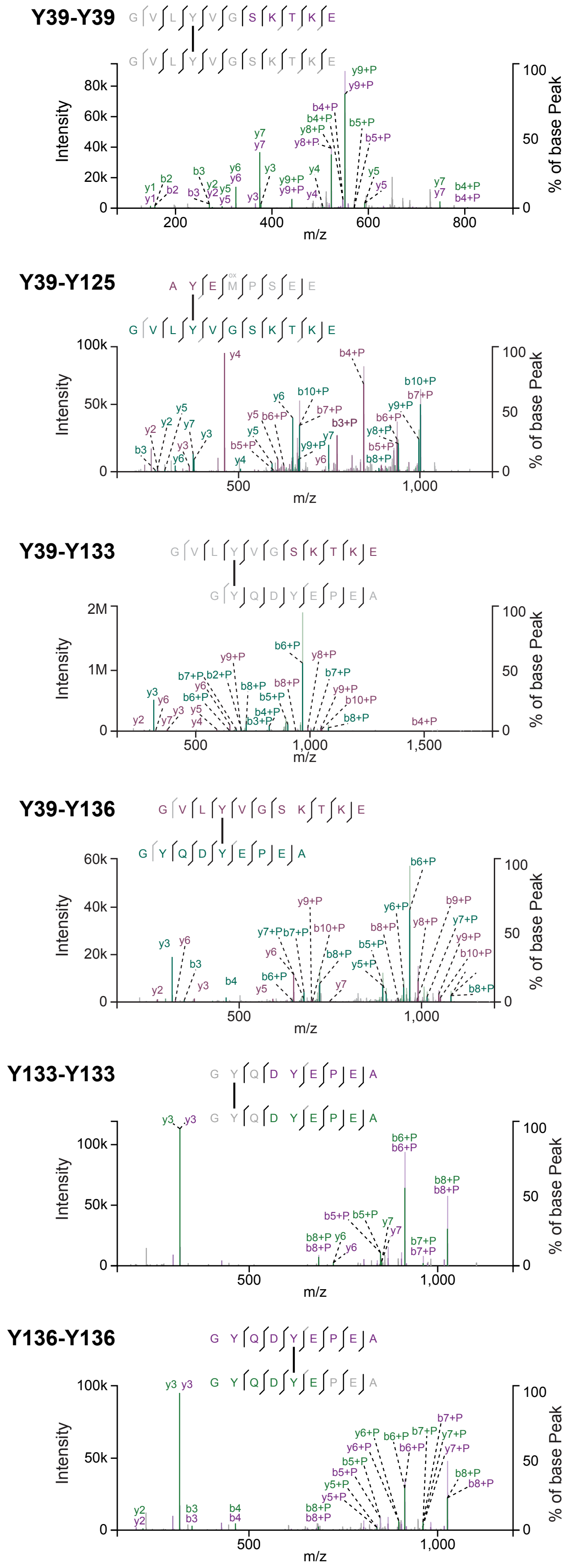

(b)

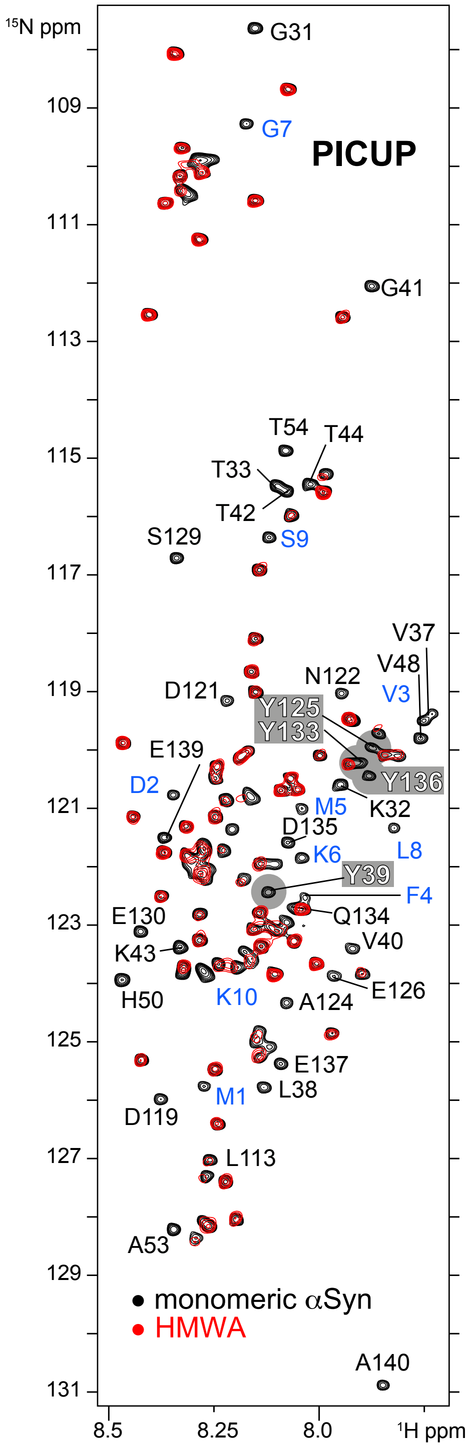

Figure S2

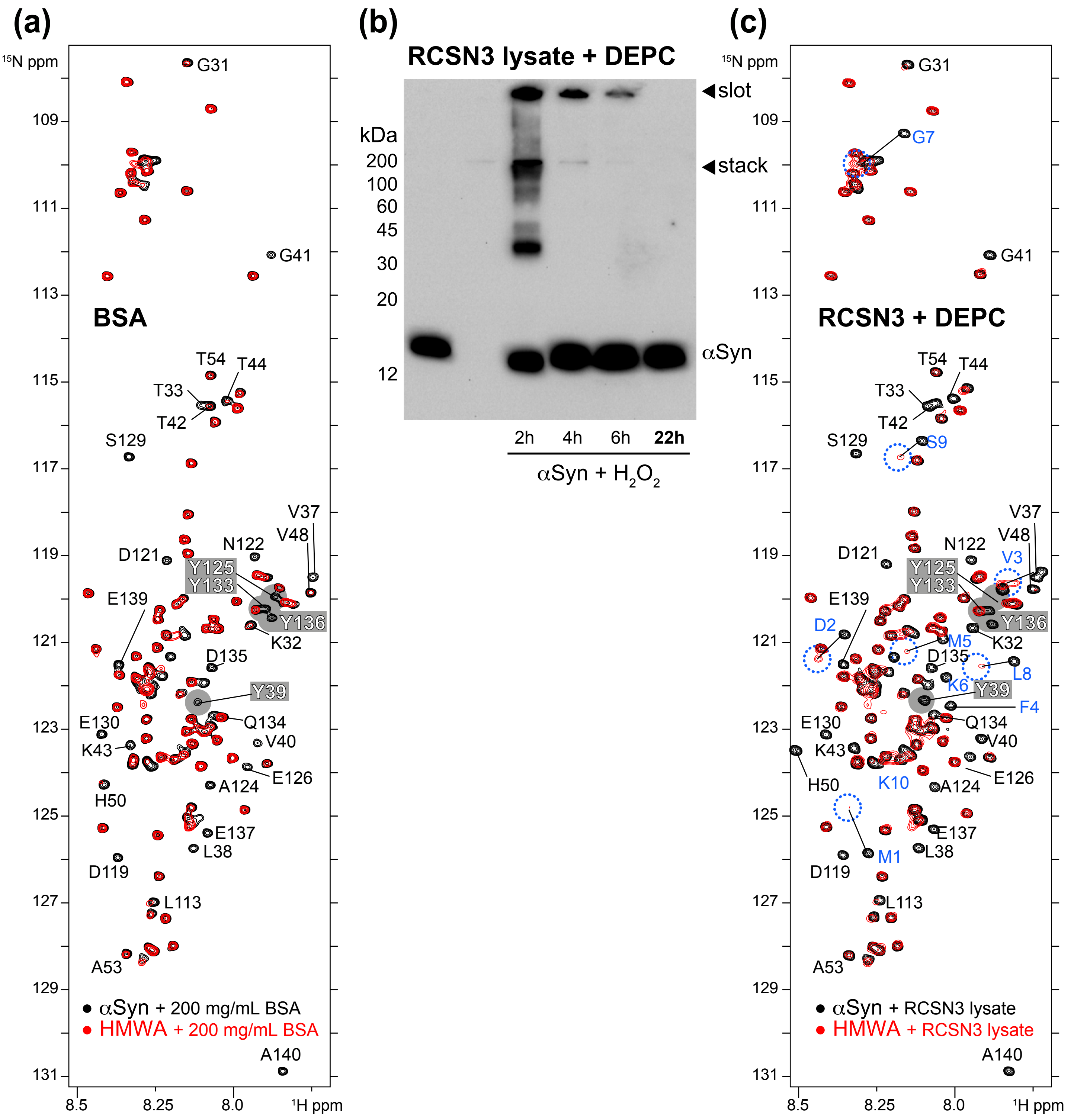
(a)
BSA
¹⁵N ppm
¹H ppm
G31
G41
T54
T44
T33
T42
S129
V37
V48
N122
D121
Y125
Y133
Y136
E139
K32
D135
Y39
E130
Q134
K43
V40
E126
A124
H50
E137
L38
D119
L113
A53
A140
● αSyn + 200 mg/mL BSA
● HMWA + 200 mg/mL BSA
(b)
RCSN3 lysate + DEPC
kDa
200
100
60
45
30
20
12
◀slot
◀stack
αSyn
2h
4h
6h
22h
αSyn + H₂O₂
(c)
RCSN3 + DEPC
G7
S9
V3
D2
M5
L8
K6
F4
K10
M1
● αSyn + RCSN3 lysate
● HMWA + RCSN3 lysate

Figure S3 Verzini *et al.*

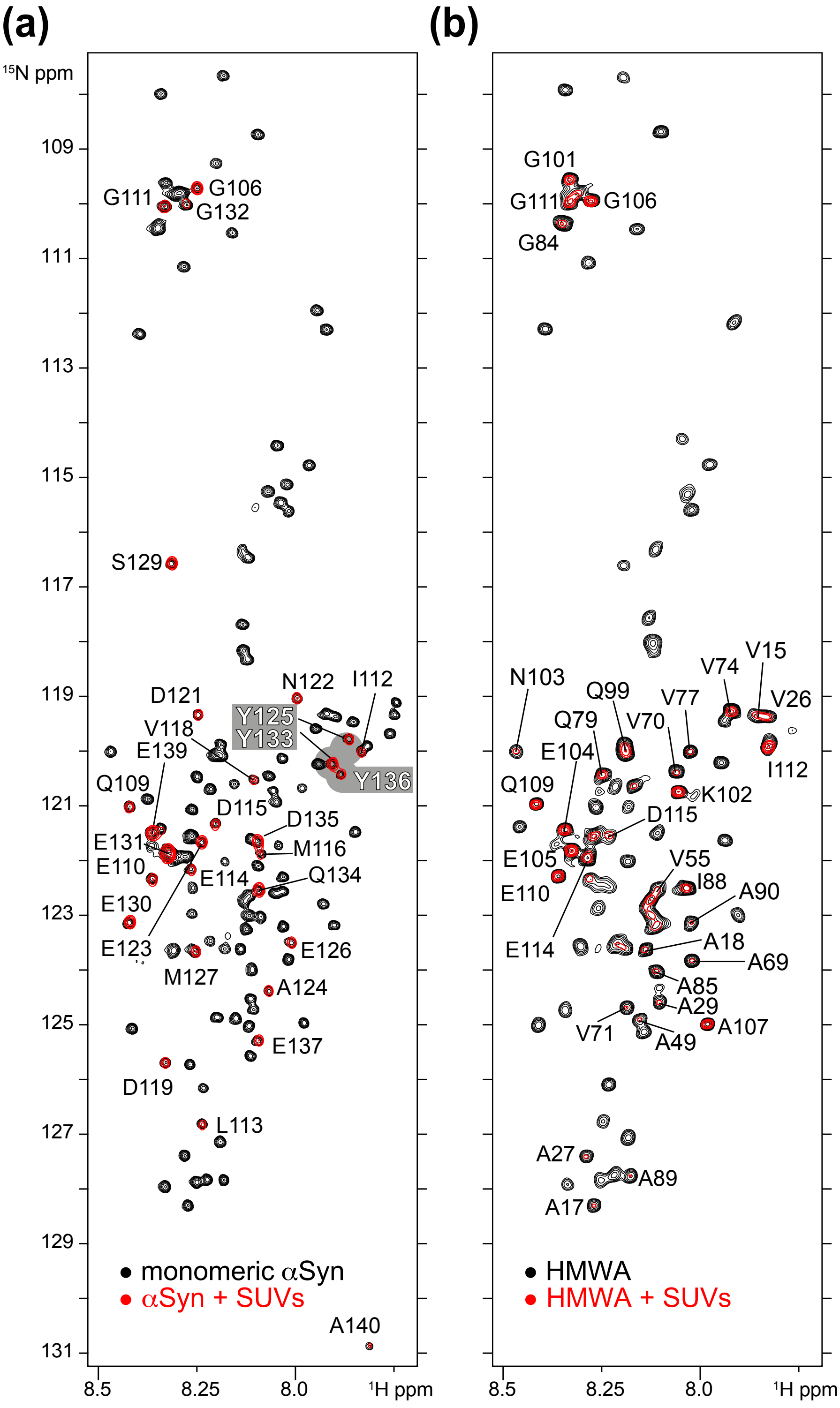

Supplement: Supplementary data 1 [file mmc1.pdf]
